# Population genetics of *Aedes albopictus* (Diptera: Culicidae) in its native range in Lao People’s Democratic Republic

**DOI:** 10.1186/s13071-019-3740-0

**Published:** 2019-10-14

**Authors:** Maysa Tiemi Motoki, Dina Madera Fonseca, Elliott Frederic Miot, Bruna Demari-Silva, Phoutmany Thammavong, Somsanith Chonephetsarath, Nothasine Phommavanh, Jeffrey Conrad Hertz, Pattamaporn Kittayapong, Paul Trevor Brey, Sebastien Marcombe

**Affiliations:** 1 Medical Entomology and Vector Borne Disease Unit, Institut Pasteur du Laos, Vientiane, Lao People’s Democratic Republic; 2 Vysnova Partners Inc., Landover, MD USA; 3 Walter Reed Biosystematics Unit, Smithsonian Institution, Museum Support Center, MRC-534, Suitland, MD 20746, USA; 4 Center for Vector Biology and Department of Entomology, Rutgers University, New Brunswick, NJ USA; 5 Université Pierre et Marie Curie, Cellule Pasteur UPMC, Paris, France; 6 Insect-Virus Interactions Group, Department of Genomes and Genetics, Institut Pasteur, Paris, France; 7 Centre National de la Recherche Scientifique, Unité Mixte de Recherche 2000, Paris, France; 8 Department of Epidemiology, Faculty of Public Health, University of Sao Paulo, Sao Paulo, Brazil; 9 U.S. Naval Medical Research Unit TWO, Singapore, Singapore; 10 Mahidol University, Bangkok, Thailand

**Keywords:** *Aedes albopictus*, *cox*1 gene, Genetic population, Lao PDR

## Abstract

**Background:**

The Asian tiger mosquito, *Aedes* (*Stegomyia*) *albopictus* (Skuse) is an important worldwide invasive species and can be a locally important vector of chikungunya, dengue and, potentially, Zika. This species is native to Southeast Asia where populations thrive in both temperate and tropical climates. A better understanding of the population structure of *Ae. albopictus* in Lao PDR is very important in order to support the implementation of strategies for diseases prevention and vector control. In the present study, we investigated the genetic variability of *Ae. albopictus* across a north-south transect in Lao PDR.

**Methods:**

We used variability in a 1337-bp fragment of the mitochondrial cytochrome *c* oxidase subunit 1 gene (*cox*1), to assess the population structure of *Ae. albopictus* in Lao PDR. For context, we also examined variability at the same genetic locus in samples of *Ae. albopictus* from Thailand, China, Taiwan, Japan, Singapore, Italy and the USA.

**Results:**

We observed very high levels of genetic polymorphism with 46 novel haplotypes in *Ae. albopictus* from 9 localities in Lao PDR and Thailand populations. Significant differences were observed between the Luangnamtha population and other locations in Lao PDR. However, we found no evidence of isolation by distance. There was overall little genetic structure indicating ongoing and frequent gene flow among populations or a recent population expansion. Indeed, the neutrality test supported population expansion in Laotian *Ae. albopictus* and mismatch distribution analyses showed a lack of low frequency alleles, a pattern often seen in bottlenecked populations. When samples from Lao PDR were analyzed together with samples from Thailand, China, Taiwan, Japan, Singapore, Italy and the USA, phylogenetic network and Bayesian cluster analysis showed that most populations from tropical/subtropical regions are more genetically related to each other, than populations from temperate regions. Similarly, most populations from temperate regions are more genetically related to each other, than those from tropical/subtropical regions.

**Conclusions:**

*Aedes albopictus* in Lao PDR are genetically related to populations from tropical/subtropical regions (i.e. Thailand, Singapore, and California and Texas in the USA). The extensive gene flow among locations in Lao PDR indicates that local control is undermined by repeated introductions from untreated sites.

## Background

Dengue fever, the potentially deadly outcome of infection with a mosquito borne flavivirus (DENV, *Flaviviridae*, *Flavivirus*), is one of the most challenging public health problems in the Greater Mekong Subregion (GMS) composed of Cambodia, China, Myanmar, Thailand, Vietnam and Lao People’s Democratic Republic (PDR) [1, 2]. From 2009 to 2012, dengue was reported in all provinces in Lao PDR, except for Phongsaly and Huaphanh provinces in northern region [3]. All four serotypes of dengue flaviviruses (DENV1-4) now circulate in rural and urban areas in Lao PDR [3–7]. In Lao PDR, an extensive dengue outbreak, mostly attributed to serotype 3 (DENV3) in 2013, caused 44,098 cases and 95 deaths [8, 9]. Again in 2017, 9832 cases of dengue fever were reported in Lao PDR, including 14 deaths, with the most affected provinces being Vientiane Capital and Champasak [10]. Both *Aedes* (*Stegomyia*) *aegypti* (Linnaeus) and *Aedes* (*Stegomyia*) *albopictus* (Skuse, 1894) were suspected to have been involved in these epidemics [11, 12]. However, still there is no study proving their vector status in the country.

*Aedes albopictus*, the Asian tiger mosquito, is thought to be native to Southeast Asia [13]. In recent decades, *Ae. albopictus* has spread throughout the world and is now found on all continents except Antarctica [14–16]; it is considered one of the most invasive and widespread mosquito species in the world [14, 17]. Despite *Ae. albopictus* being considered a secondary vector of dengue and chikungunya (CHIKV, *Togaviridae*, *Alphavirus*) relative to *Ae. aegypti* [18], in some instances such as in central Africa, China and Mediterranean Europe [19–21] it can become the primary vector. Of note, several laboratory studies have shown that *Ae. albopictus* can be more competent at transmitting DENV and CHIKV than *Ae. aegypti* [22–24]. Furthermore, *Ae. albopictus* has been associated with the emergence of Zika virus from its native Africa, although this is still in early stages of investigation [25–27].

Although mosquito populations with different genetic makeup may differ in vector competence [28], there is currently no information about the population genetics of *Ae. albopictus* in Lao PDR. Information about genetic diversity and population structure can be a tool in the development of effective mosquito control programmes [29, 30]. Therefore, we obtained samples of *Ae. albopictus* from eight provinces from the northwest, northern, central and southern regions of Lao PDR including the two most affected provinces, Vientiane Capital and Champasak, and sequenced a fragment of the cytochrome *c* oxidase subunit 1 gene (*cox*1) mitochondrial (mt) DNA. First, we analyzed the genetic variability of samples from Lao PDR, then compared against other samples from China, Japan, Taiwan, Singapore, the USA, Italy [31] and Thailand to check the genetic relationships among them. Our primary aim was to increase our understanding of the population structure of *Ae. albopictus* in Laos in order to develop better strategies for dengue prevention and vector control in Lao PDR.

## Methods

### Mosquito collection and identification

**Fig. 1:**
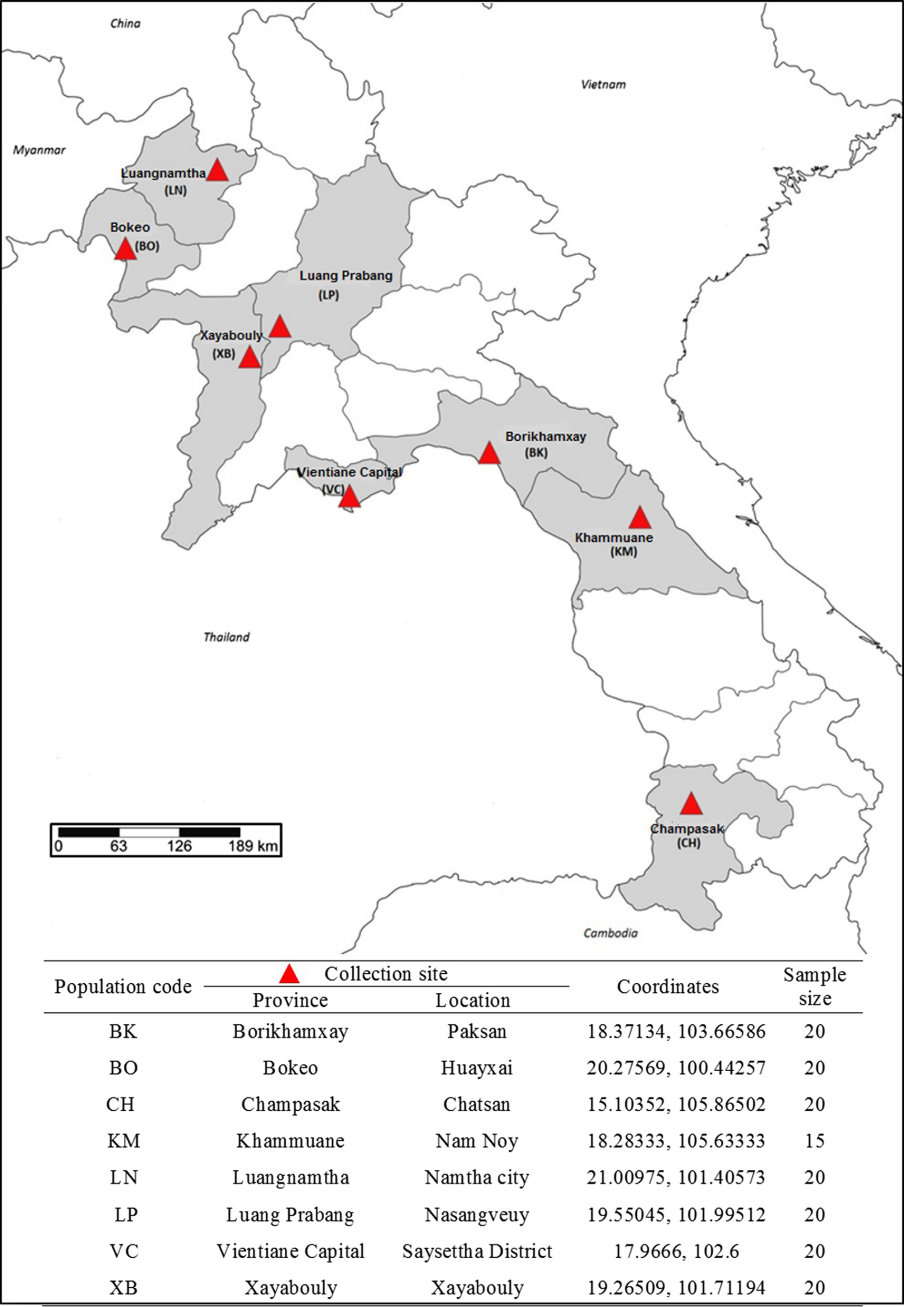
Collection information of *Aedes albopictus* in Lao PDR

The collections were carried out in eight localities from the northwest [Bokeo (BK), Luangnamtha (LN) and Xayabouly (XB) Provinces], northern [Luang Prabang (LP) Province], central [Vientiane prefecture (VC), Borikhamxay (BK) and Khammuane (KM) Provinces] and southern [Champasak (CH) Province] regions of Lao PDR (Fig. 1). *Aedes albopictus* larvae and pupae were collected between 2014 and 2016 from domestic containers (tanks and jars) and peri-domestic habitats (used tires, discarded containers, etc.), then carefully transferred into WhirlPak plastic bags (BioQuip, Rancho Dominguez, CA, USA) and sent to the insectaries in Vientiane for rearing (field generation, F0). Each mosquito population sample consisted of larvae and pupae collected from at least 10 breeding sites per locality to reduce the likelihood of re-sampling them. Female mosquitoes were then stored individually in a desiccated tube at − 80 °C until molecular analyses. All mosquitoes were morphologically identified as *Ae. albopictus* using available keys [32] and confirmed by comparison of *cox*1 barcode region sequences available on GenBank.

### DNA extraction and sequencing

Total genomic DNA was extracted from single whole mosquitoes using a NucleoSpin^®^ Tissue kit (Macherey-Nagel, Duren, Germany) according to manufacturer’s instructions. The fragment of mtDNA cytochrome *c* oxidase subunit 1 (*cox*1) gene was amplified using two sets of primers, 1454F (5′-GGT CAA CAA ATC ATA AAG ATA TTG G-3′) and 2160R (5′-TAA ACT TCT GGA TGA CCA AAA AAT CA-3′); and 2027F (5′-CCC GTA TTA GCC GGA GCT AT-3′) and 2886R (5′-ATG GGG AAA GAA GGA GTT CG-3′), following the polymerase chain reaction (PCR) protocol explicitly detailed in Zhong et al. [31]. Aliquots of the PCR products were visualized on 1.5% agarose gels and successful amplifications were purified using ExosapIT^®^ (USB Co, Cleveland, OH, USA). All sequencing reactions were carried out in both directions using an ABI Big Dye Terminator Kit v.3.1 (Applied Biosystems, Warrington, UK) and analyzed on an ABI Prism 3500xL—Avant Genetic Analyzer (Applied Biosystems, Foster City, CA, USA) at the Institut Pasteur du Laos sequencing facilities in Vientiane.

### Data analyses

The *cox*1 gene sequences were edited using Sequencher^®^ version 5.4.6 (Gene Codes Corporation, Ann Arbor, MI, USA) and automatically aligned in Geneious v.9.1.6. [33].

The number of haplotypes (H), haplotype diversity (*Hd*), nucleotide diversity (π) and (*K*) average of nucleotide differences within each site were generated using DnaSP v.5.0 [34]. The pairwise *F*_*ST*_ was calculated to estimate population differentiation based on differences in haplotype frequencies, whereas Nei’s *Nm* estimated gene flow is based on *G*_*ST*_ [35] using Arlequin v.3.5 [36].

Analysis of molecular variance (AMOVA) was conducted to determine the distribution of genetic variation within and among populations using 1000 permutations implemented in Arlequin v.3.5 [36]. Additionally, a spatial analysis of molecular variance (SAMOVA) v.1.0 [37] was used to cluster the 1337-bp *cox*1 sequences into genetically and geographically homogeneous populations. SAMOVA generates *F*-statistics (*F*_*SC*_, *F*_*ST*_, *F*_*CT*_), using the AMOVA approach, into *K* groups to maximize the between group variation. SAMOVA estimates were computed for *K* = 2–8, with 1000 simulated annealing steps from each of 100 sets of initial starting conditions. Isolation by distance (IBD) was checked using a Mantel tests [38]. IBD was estimated in GenAlEx v.6.5 [39, 40] between the genetic and geographical distances with 10,000 permutations.

The hypothesis of strict neutrality among *Ae. albopictus* populations from Lao PDR was examined using the statistics *D* [41] and Fu’s *F*_*S*_ [42], calculated using DnaSP v.5.0 [34]. The mismatch distribution (simulated in Arlequin v.3.5) was performed to distinguish between a smooth unimodal distribution and a multimodal or ragged distribution [43–45]. Statistically significant differences between observed and simulated distributions were evaluated with the sum of square deviations (SSD) to reject the hypothesis of demographic expansion [46].

To make broader comparisons among haplotypes from Lao PDR and other geographical regions, we analyzed samples from Thailand and downloaded available data in GenBank from China, Taiwan, Japan, Singapore, Italy and the USA [31]. The parsimony network was performed using TCS network inference method [47] in Population Analysis with Reticulate Trees (PopART) [48]. We also checked the number of haplotypes, *F*_*ST*_, *Nm* and AMOVA using the same methodology described earlier.

In addition, a Bayesian clustering algorithm implemented in the program STRUCTURE v.2.3 was used to investigate genetic structure of individuals. The program was run under varying assumptions on Hardy-Weinberg (HW) and linkage equilibriums [49], with ten independent runs performed for each value of *K* (*K* = 1 to 21). In this analysis, the most likely number of genetic clusters (*K*) in the dataset is determined without prior information of the sampling locations, and then assigns proportion of the ancestry of each individual into the different clusters implemented in the program. The method of Evanno et al. [50] was used to determine the most likely number of clusters. This approach uses an *ad hoc* quantity, based on the second rate of change of the likelihood function between successive values of *K*. Poterior probability values were estimated using a Markov Chain Monte Carlo (MCMC) method and 1,000,000 interactions of each chain following the 100,000 iteration burn-in period were performed, as recommended by Pritchard et al. [49]. We visualized the partitioning of clusters using the program DISTRUCT [51].

## Results

### Genetic diversity

Partial sequences of the mtDNA *cox*1 (1337-bp) were amplified from 172 specimens, representing populations from Lao PDR (*n* = 155) and Thailand (*n* = 17). No insertions, deletions or stop codons were detected across all samples, which minimizes the likelihood of pseudogene amplification.

A total of 44 haplotypes were identified among the Lao populations (Table 1); of these, 13 haplotypes (30%) were shared among Lao populations and 31 (70%) were unique to single Lao populations. When the data was combined with Zhong et al. [31] (H1–H66) and the Thailand samples, a total of 46 haplotypes were found. These newly identified haplotypes are H67–H112 and were deposited in GenBank under accession numbers MN080720–MN080765 (Table 1). Lao PDR sequences shared five haplotypes with Thailand, two haplotypes (H45 and H56) with the USA (California and Texas, respectively) and Thailand, and one haplotype (H46) with the USA (California) (Table 1).

**Table 1:** Haplotypes of *Aedes albopictus* based on the mtDNA *cox*1 marker

Haplotype	*n*	Country (code)	GenBank ID
H01^a^	6	China (GZ, XM), USA (LA01)	KC690896
H02^a^	5	China (GZ)	KC690897
H03^a^	113	China (GZ, XM, JS,), Taiwan (TW), Japan (JP), Italy (IT), USA (LA01, LA11, HW)	KC690898
H04^a^	3	China (GZ)	KC690899
H05^a^	1	China (GZ)	KC690900
H06^a^	2	China (GZ)	KC690901
H07^a^	1	China (XM)	KC690902
H08^a^	9	China (XM)	KC690903
H09^a^	1	China (XM)	KC690904
H10^a^	1	China (XM)	KC690905
H11^a^	1	China (XM)	KC690906
H12^a^	2	China (XM)	KC690907
H13^a^	2	China (XM)	KC690908
H14^a^	1	China (XM)	KC690909
H15^a^	1	China (XM)	KC690910
H16^a^	7	China (JS)	KC690911
H17^a^	26	Taiwan (TW), Italy (IT), USA (LA11, TX, HW)	KC690912
H18^a^	1	Taiwan (TW)	KC690913
H19^a^	2	Taiwan (TW), USA (LA11)	KC690914
H20^a^	1	Taiwan (TW)	KC690915
H21^a^	1	Taiwan (TW)	KC690916
H22^a^	3	Taiwan (TW), USA (LA11)	KC690917
H23^a^	1	Taiwan (TW)	KC690918
H24^a^	23	Japan (JP), Singapore (SG)	KC690919
H25^a^	1	Japan (JP)	KC690920
H26^a^	1	Singapore (SG)	KC690921
H27^a^	8	Singapore (SG)	KC690922
H28^a^	1	Singapore (SG)	KC690923
H29^a^	1	Singapore (SG)	KC690924
H30^a^	2	Singapore (SG)	KC690925
H31^a^	2	Singapore (SG)	KC690926
H32^a^	1	Singapore (SG)	KC690927
H33^a^	1	Singapore (SG)	KC690928
H34^a^	1	Singapore (SG)	KC690929
H35^a^	1	Singapore (SG)	KC690930
H36^a^	1	Italy (IT)	KC690931
H37^a^	40	Italy (IT), USA (NJ, TX)	KC690932
H38^a^	1	Italy (IT)	KC690933
H39^a^	6	Italy (IT), USA (TX)	KC690934
H40^a^	2	Italy (IT)	KC690935
H41^a^	4	Italy (IT)	KC690936
H42^a^	1	Italy (IT)	KC690937
H43^a^	1	Italy (IT)	KC690938
H44^a^	1	Italy (IT)	KC690939
H45^a, b^	52	USA (LA01), Laos (BK, BO, CH, KM, LN, LP, VC, XB), Thailand (TH)	KC690940
H46^a, b^	5	USA (LA01), Laos (BK, BO, CH)	KC690941
H47^a^	1	USA (LA01)	KC690942
H48^a^	2	USA (LA01)	KC690943
H49^a^	7	USA (LA11)	KC690944
H50^a^	1	USA (LA11)	KC690945
H51^a^	2	USA (NJ)	KC690946
H52^a^	4	USA (NJ)	KC690947
H53^a^	2	USA (NJ)	KC690948
H54^a^	2	USA (NJ)	KC690949
H55^a^	3	USA (TX)	KC690950
H56^a, b^	5	USA (TX), Laos (BK, BO), Thailand (TH)	KC690951
H57^a^	1	USA (TX)	KC690952
H58^a^	1	USA (TX)	KC690953
H59^a^	1	USA (TX)	KC690954
H60^a^	1	USA (TX)	KC690955
H61^a^	16	USA (HW)	KC690956
H62^a^	2	USA (HW)	KC690957
H63^a^	1	USA (HW)	KC690958
H64^a^	1	USA (HW)	KC690959
H65^a^	1	USA (HW)	KC690960
H66^a^	1	USA (HW)	KC690961
H67^c^	1	Lao PDR (BK)	MN080720
H68^c^	1	Lao PDR (BK)	MN080721
H69^c^	4	Lao PDR (BK, CH), Thailand (TH)	MN080722
H70^c^	1	Lao PDR (BK)	MN080723
H71^c^	11	Lao PDR (BK, BO, LP)	MN080724
H72^c^	1	Lao PDR (BK)	MN080725
H73^c^	1	Lao PDR (BK)	MN080726
H74^c^	1	Lao PDR (BK)	MN080727
H75^c^	6	Lao PDR (BO, CH, LN)	MN080728
H76^c^	20	Lao PDR (BO, LN, XB), Thailand (TH)	MN080729
H77^c^	4	Lao PDR (BO)	MN080730
H78^c^	2	Lao PDR (CH)	MN080731
H79^c^	1	Lao PDR (CH)	MN080732
H80^c^	1	Lao PDR (CH)	MN080733
H81^c^	1	Lao PDR (CH)	MN080734
H82^c^	1	Lao PDR (CH)	MN080735
H83^c^	1	Lao PDR (CH)	MN080736
H84^c^	3	Lao PDR (CH, LP, VC)	MN080737
H85^c^	2	Lao PDR (KM)	MN080738
H86^c^	6	Lao PDR (KM, VC)	MN080739
H87^c^	4	Lao PDR (KM, VC)	MN080740
H88^c^	1	Lao PDR (KM)	MN080741
H89^c^	2	Lao PDR (KM)	MN080742
H90^c^	3	Lao PDR (KM, XB)	MN080743
H91^c^	1	Lao PDR (LP)	MN080744
H92^c^	2	Lao PDR (LP)	MN080745
H93^c^	2	Lao PDR (LP, VC)	MN080746
H94^c^	1	Lao PDR (LP)	MN080747
H95^c^	1	Lao PDR (LP)	MN080748
H96^c^	2	Lao PDR (LP), Thailand (TH)	MN080749
H97^c^	1	Lao PDR (LP)	MN080750
H98^c^	2	Lao PDR (VC)	MN080751
H99^c^	2	Lao PDR (VC)	MN080752
H100^c^	1	Lao PDR (VC)	MN080753
H101^c^	1	Lao PDR (VC)	MN080754
H102^c^	2	Lao PDR (VC)	MN080755
H103^c^	1	Lao PDR (VC)	MN080756
H104^c^	1	Lao PDR (VC)	MN080757
H105^c^	1	Lao PDR (VC)	MN080758
H106^c^	2	Lao PDR (XB)	MN080759
H107^c^	4	Lao PDR (XB)	MN080760
H108^c^	1	Thailand (TH)	MN080761
H109^c^	2	Thailand (TH)	MN080762
H110^c^	3	Thailand (TH)	MN080763
H111^c^	1	Thailand (TH)	MN080764
H112^c^	1	Thailand (TH)	MN080765

^a^Haplotype data obtained from Zhong et al. [31]

^b^Shared haplotypes with Zhong et al. [31]

^c^New haplotypes

*Abbreviations*: GZ, Guangzhou; XM, Xiamen; JS, Jiangsu; TW, Xinzhu; JP, Nagazaki; SG, Helios Block; IT, Trentino; LA01, California; LA11, California; NJ, New Jersey; TX, Texas; HW, Hawaii; BK, Borikhamxay; BO, Bokeo; CH, Champasak; KM, Khammuane; LN, Luangnamtha; LP, Luang Prabang; VC, Vientiane Capital; XB, Xayabouly; TH, Thailand

Zhong et al. [31] amplified a fragment of 1433-bp of the mtDNA *cox*1, and identified 66 haplotypes of *Ae. albopictus* in 6 different countries (Italy, Japan, Taiwan, China, Singapore and the USA; 12 populations). The trimmed fragment we used is 96-bp smaller (1337-bp) than that of Zhong et al. [31]; however, no polymorphic sites were included in the trimmed sequence. Therefore, when we trimmed all fragments to 1377-bp, we still have the same 66 haplotypes as Zhong et al. [31] and 46 new haplotypes were recognized, totaling 112 haplotypes (Table 1).

The average number of nucleotide differences in *Ae. albopictus* in Lao PDR populations ranged from 0.537 (LN) to 3.105 (KM), corresponding with the range of the nucleotide diversity (π) 0.00040 (LN) to 0.00232 (KM). Haplotype diversity (*Hd*) ranged from 0.416 ± 0.116 (mean ± SD) (LN) to 0.942 ± 0.029 (VC) (Table 2).

**Table 2:** Summary of haplotype and nucleotide diversity measures of the *cox*1 gene for *Ae. albopictus* in Lao PDR

Population code	*n*	H	*Hd* ± SD	π ± SD	*K*
BK	20	11	0.874 ± 0.064	0.00150 ± 0.0009	2.000
BO	20	7	0.821 ± 0.056	0.00131 ± 0.0009	1.753
CH	20	11	0.805 ± 0.090	0.00110 ± 0.0008	1.468
KM	15	8	0.914 ± 0.043	0.00232 ± 0.0010	3.105
LN	20	3	0.416 ± 0.116	0.00040 ± 0.0004	0.537
LP	20	11	0.868 ± 0.064	0.00145 ± 0.0009	1.942
VC	20	12	0.942 ± 0.029	0.00220 ± 0.0012	2.937
XB	20	5	0.716 ± 0.087	0.00110 ± 0.0007	1.474

*Abbreviations*: n, number of individuals analyzed; H, number of haplotypes; *Hd*, haplotype diversity; SD, standard deviation; π, nucleotide diversity; *K*, average of nucleotide differences; BK, Borikhamxay; BO, Bokeo; CH, Champasak; KM, Khammuane; LN, Luangnamtha; LP, Luang Prabang; VC, Vientiane Capital; XB, Xayabouly

The highest level of genetic differentiation in Lao PDR based on the fixation index *F*_*ST*_ was between LN and LP (*F*_*ST*_ = 0.33288, *P* ˂ 0.05). Gene flow (*Nm*) was > 1 among all populations, except LN and XB (Table 3). When analyzed all together including the samples of Zhong et al. [31], the highest *F*_*ST*_ was between LN (Luangnamtha, Lao PDR) and JS (Jiangsu, China) (*F*_*ST*_ = 0.610, *P* ˂ 0.05) (Additional file 1: Table S1).

**Table 3:** Pairwise differentiation (*F*_*ST*_, below the diagonal), and gene flow (*N*_*m*_, above the diagonal) among populations of *Ae. albopictus* in Lao PDR

	BK	BO	CH	KM	LN	LP	VC	XB
BK	–	6.50505	∞	12.2500	3.47518	5.69707	6.08466	2.33385
BO	0.07138*	–	4.42693	4.62039	1.51515	3.83055	4.06553	10.50360
CH	− 0.00838	0.10148**	–	8.32182	7.27273	3.02857	4.20786	1.95535
KM	0.04122	0.09765**	0.05668*	–	1.65733	4.48241	25.51247	2.50000
LN	0.12578**	0.24812**	0.06433	0.23177**	–	1.00205	1.19722	0.84646
LP	0.08068*	0.11546**	0.14170**	0.10035**	0.33288**	–	5.04399	1.95201
VC	0.07593**	0.10952**	0.10621**	0.01922	0.29460**	0.09019**	–	2.49604
XB	0.17644**	0.04544	0.20364**	0.16667**	0.37135**	0.20391**	0.16689*	–

* Significant values after Bonferroni correction (* *P* ˂ 0.05, ** *P* ˂ 0.01)

*Abbreviation*s: BK, Borikhamxay; BO, Bokeo; CH, Champasak; KM, Khammuane; LN, Luangnamtha; LP, Luang Prabang; VC, Vientiane Capital; XB, Xayabouly

Global AMOVA tests indicated a high proportion of the total genetic variance was attributable to within-population variation (85.98%), suggesting low and significant genetic structure among populations (*F*_*ST*_ = 0.14, *P* ≤ 0.001) in Lao PDR. When we added all samples including that of Zhong et al. [31], global AMOVA found a significant overall population structure in *Ae. albopictus* (*F*_*ST*_ = 0.43, *P* ≤ 0.001), with 56.8% of genetic variation found within-population and 43.2% among-populations. The spatial analysis of molecular variance (SAMOVA), based on mtDNA data, showed no genetically distinct population groups. Partitions of the sampling areas for each *K* value were not informative. *F*_*CT*_ values presented a narrow range between 0.18 and 0.23. (Additional file 2: Figure S1). Mantel tests showed that genetic and geographical distances (Additional file 3: Table S2) among populations in Lao PDR do not support a pattern of isolation by distance (*r* = 0.0846, *P* = 0.1433).

Assessment of population expansion based on neutrality test resulted primarily in negative values but most were not statistically significant, with the exception of Tajima’s *D* for CH, and Fu’s *Fs* for BK, CH, LN and LP (Table 4). Mismatch distribution models revealed poor fit to equilibrium distribution (Additional file 4: Figure S2); both the sum of squared deviation (SSD) values (0.016, *P* = 0.29) and raggedness index (0.09) were not statistically significant in almost all the populations, except the SSD value for BO and CH and Rag for CH (Table 4), indicating further support for population expansion based on *cox*1 gene.

**Table 4:** Neutrality test and mismatch distribution of *cox*1 gene of *Ae. albopictus* in Lao PDR

Population code	Neutrality tests		Mismatch analysis	
	*D*	*F*_*S*_	SSD	Rag
BK	− 1.25890	− 6.08335*	0.012	0.056
BO	0.11573	− 1.61256	0.040*	0.144
CH	− 2.03130*	− 8.03746*	0.017*	0.131*
KM	− 1.10557	− 1.58578	0.017	0.059
LN	− 0.97524	− 0.07875*	0.000	0.132
LP	0.47276	− 6.25889*	0.008	0.046
VC	0.54226	− 5.33883	0.007	0.038
XB	0.88892	− 0.12444	0.028	0.144
Mean	− 0.41892	− 3.64001	0.016	0.09

* Significant value, *P* ˂ 0.01

*Abbreviations*: BK, Borikhamxay; BO, Bokeo; CH, Champasak; KM, Khammuane; LN, Luangnamtha; LP, Luang Prabang; VC, Vientiane Capital; XB, Xayabouly

### Genetic relationships among haplotypes

**Fig. 2:**
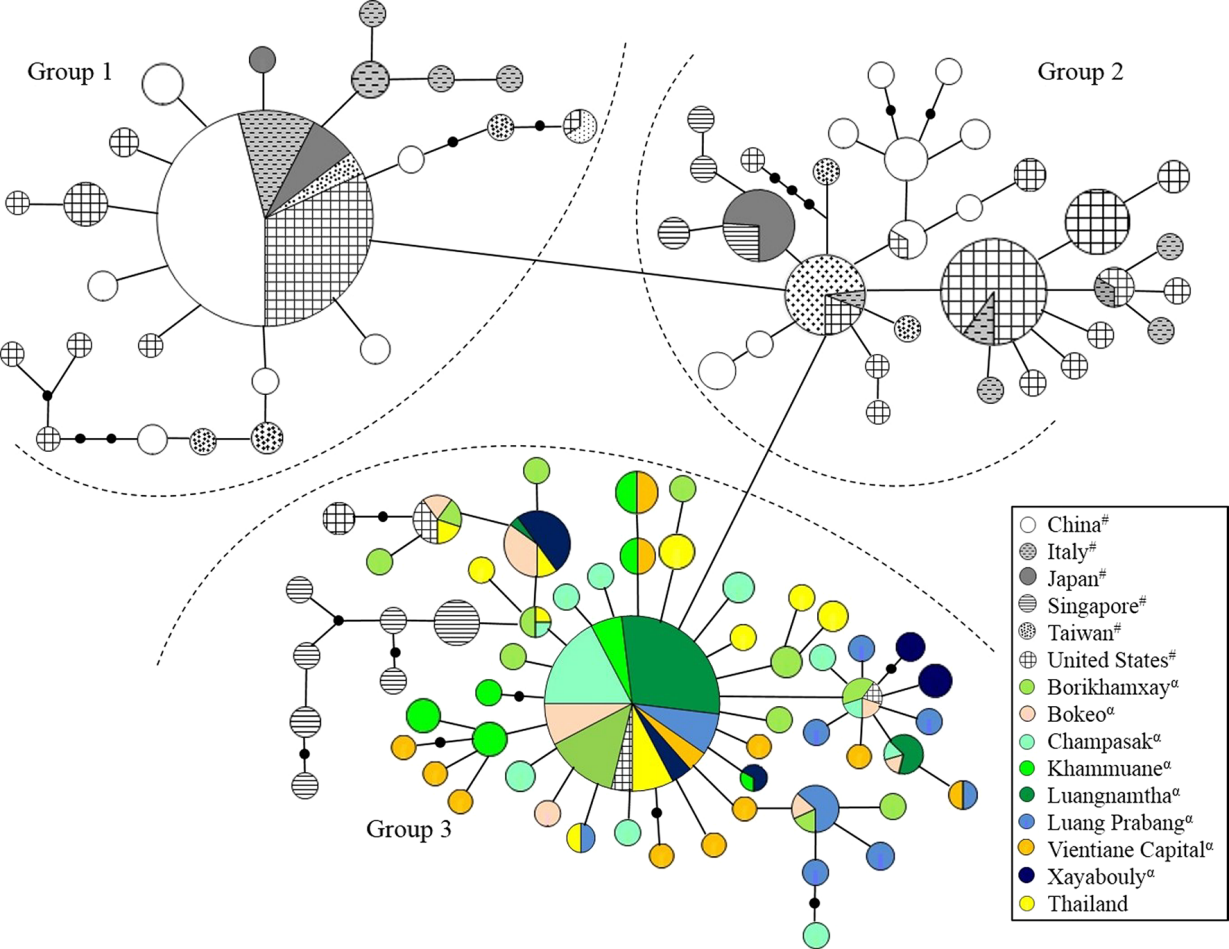
Phylogenetic network of 112 mitochondrial haplotypes (1337 bp) of the *cox*1 gene in *Ae. albopictus*. Localities are indicated by different colors (bottom-right). The area of each circle is approximately proportional to the frequency of the haplotype. ^#^Samples available in Genbank from Zhong et al. [31]. ^α^Samples from Lao PDR

The parsimony network showed that the genealogical relationships among the haplotypes differed by 4–9 mutational steps (Fig. 2) and can be divided into three Groups: Group 1 mainly contained haplotypes from China, and a number of haplotypes from Japan, Italy, Taiwan and the USA; Group 2 contained haplotypes from China, Japan, Italy, Taiwan, the USA, and 50% of the haplotypes in Singapore; and Group 3 contained haplotypes from Lao PDR, Thailand, the remaining 50% from Singapore, and three haplotypes shared with the USA. The most common haplotypes were 3 (*n* = 113) and 45 (*n* = 52) (Fig. 2, Table 2). Haplotype 3 was shared among populations from China, Taiwan, Japan, Italy and the USA, while H45 was shared among the USA, Thailand and all populations from Lao PDR (Fig. 2, Table 1).

### Genetic clustering of individuals

**Fig. 3:**
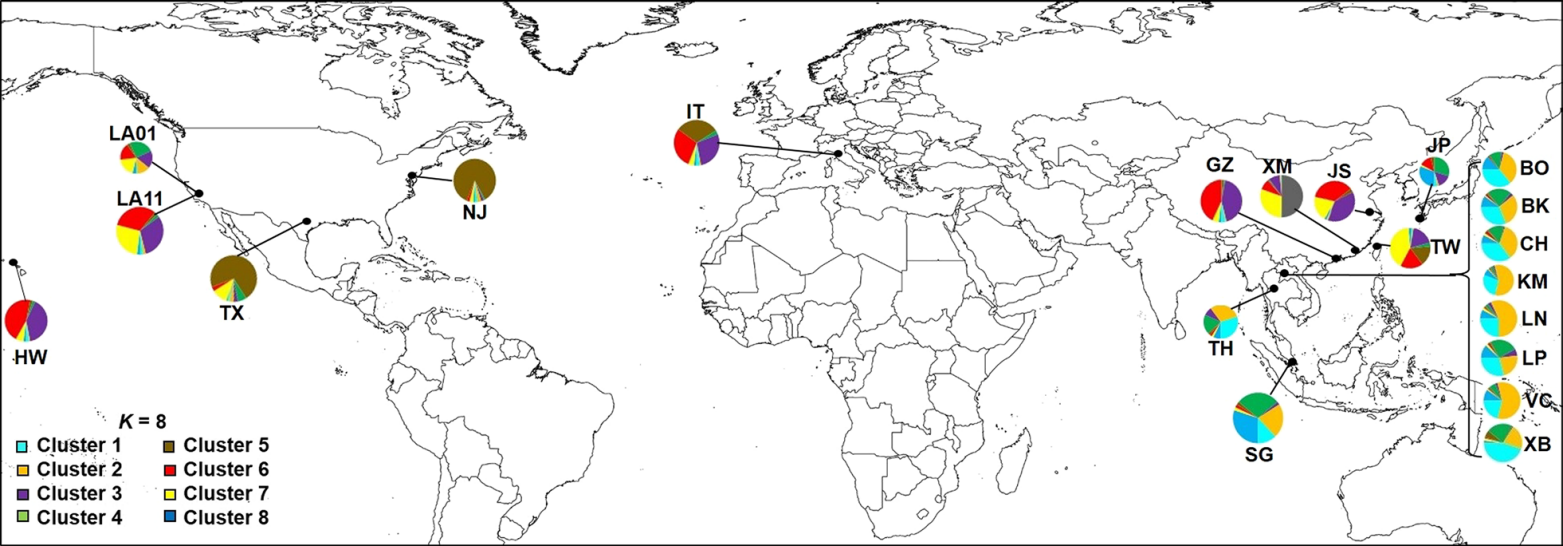
Pie charts representing the proportional membership of *Ae. albopictus* identified in Bayesian cluster analysis (optimal partitioning of all samples, *K* = 8). *Abbreviations*: China: GZ, Guangzhou; XM, Xiamen; JS, Jiangsu; Taiwan: TW, Xinzhu; Japan: JP, Nagazaki; Singapore: SG, Helios Block; Italy: IT, Trentino; USA: LA01, California; LA11, California; NJ, New Jersey; TX, Texas; and HW, Hawaii – are samples from Zhong et al. [31]; Lao PDR: BK, Borikhamxay; BO, Bokeo; CH, Champasak; KM, Khammuane; LN, Luangnamtha; LP, Luang Prabang; VC, Vientiane Capital; XB, Xayabouly; Thailand: TH, Thailand

Bayesian inference implemented in STRUCTURE revealed that the optimal partitioning of all *Ae*. *albopictus* samples (China, Taiwan, Japan, Singapore, Italy, USA from Zhong et al. [31], Lao PDR and Thailand) was *K* = 8. The individuals analyzed from the 21 populations were assigned to eight clusters with a certain probability value (Fig. 3, Additional file 5: Table S3). Most individuals from Lao PDR and Thailand were represented in clusters 1 and 2, and partially in clusters 4 and 8, sharing with Singapore, Japan and the USA (California samples). Samples from China were mainly found in clusters 3, 6 and 7, sharing with USA and Italy, and cluster 5 included the highest proportion of individuals from the USA (New Jersey and Texas samples: 86 and 72%, respectively), as observed in Zhong et al. [31].

## Discussion

The barcoding mitochondrial gene, *cox*1, has been widely used to analyze genetic diversity in *Ae. albopictus* [31, 52–59]. In this study, we followed the recommendation of Goubert et al. [60] that reviewed the literature on the use of the *cox*1 for population genetic studies, and employed a longer mtDNA marker designed by Zhong et al. [31].

### Genetic diversity in *Ae. albopictus* from Lao PDR

Overall, we detected very high haplotype diversity in *Ae. albopictus* in Lao PDR, with 44 haplotypes identified from only eight populations. Among them, 13 haplotypes were shared (Table 1), in some cases by all eight populations. Low and significant genetic structure (Table 3) were observed, supporting the finding of other studies [55, 57, 59, 61].

The higher and significant differentiation among LN (Luangnamtha) and other locations in Lao PDR (*F*_*ST*_ 0.126–0.371), except CH (Champasak), may be due to climate (Table 3). Indeed, while Lao PDR has a predominantly tropical climate, the mountainous topography and the extensive Mekong River network in the northern and southern regions, results in variation in average temperature conditions and creates significantly different microclimates that may be highly relevant to mosquito development. On the other hand, the Mantel test revealed no correlation between genetic and geographical distances, indicating no isolation by distance of *Ae. albopictus* in Lao PDR. Similar results were observed within countries [60, 62–65], except in Schmidt et al. [66]; they analyzed genetic structure of *Ae. albopictus* from 12 localities in China using single nucleotide polymorphism (SNPs) and found evidence for IBD.

Signs of recent expansion observed in *Ae. albopictus* across Lao PDR are evidenced by economic development, which is characterized by high rates of urbanization in the Association of South East Asian Nations (ASEAN) community. This has led to a better road infrastructure throughout the country and has increased connectivity between all the provinces, which has the potential to facilitate human-assisted movement of *Aedes* mosquitoes (SM, personal observation) and their pathogens [13, 67]. In addition, rubber plantations provided several potential breeding sites for *Ae. albopictus* including latex-collection cups [68, 69]. According to Tangena et al. [12], the risk of dengue infection in natural forests and rubber plantations is higher than in northern region villages in Luang Prabang Province. *Aedes albopictus* is highly adaptable and successfully spread from its preferred forested environments to different rural and urban habitats, which has increased its potential as a vector and, consequently, arboviruses transmission risk in these more populated areas.

### Genetic relationship among *Ae. albopictus* in Lao PDR and the available haplotypes from other geographical regions and genetic clustering of individuals

When populations of *Ae. albopictus* were analyzed from many different geographical regions, three haplotypes were observed to be shared between Lao PDR and other countries. H45 and H46 were shared with the USA (California) and Thailand, and H56 with the USA (Texas). H45 and H46 are shared haplotypes from Los Angeles, California, where samples were collected in 2001. Similarly, Zhong et al. [31] observed those haplotypes were shared with Singaporean populations and were not found in their collection in 2011; hence, the authors suggested that only specimens from subtropical/temperate climates could have established successfully in the USA. In addition, the *F*_*ST*_ was lower when comparing Lao PDR with the 2001 California samples (0.093–0.323, *P* ≤ 0.05) than the ones collected 10 years later in 2011 (0.286–0.529, *P* ≤ 0.05) (Additional file 1: Table S1).

The phylogenetic network and the Bayesian cluster analyses corroborated the results from Zhong et al. [31]. Groups 1 and 2 (in the network analysis) and clusters 3, 5–7 (in the Bayesian analysis) included samples from temperate regions and most of group 3 and clusters 1, 2, 4, 8 (network and Bayesian analyses, respectively) included the majority of samples from tropical/subtropical regions (Figs. 2, 3). Allozyme studies have shown that populations of *Ae. albopictus* from Japan are likely distinct from the remaining samples in the world [70] and Southeast Asia (Borneo, peninsula Malaysia) and southern Asian populations (India, Sri Lanka) can both be differentiated from northern Asian populations (China, Japan) [71]. Worldwide mitogenome diversity of *Ae. albopictus* was studied and three major haplogroups were found; the first haplogroup was mostly distributed in tropical regions, the second in temperate regions and the third appeared to be important in the spread of *Ae. albopictus* from Asia [61]. A possible explanation for these differences is the presence of a photoperiodic diapause in *Ae. albopictus* from temperate regions [72–74], and absence of diapause among *Ae. albopictus* in tropical regions, such as in Brazil [72]. However, it is worth noting that the Singapore population represents a particular case in Southeast Asia. Its population is genetically connected both with tropical and temperate strains (Figs. 2, 3).

Although no study has performed a comprehensive analysis of the species’ full native range [60], the genetic differentiation of native Asian populations of *Ae. albopictus* may confer both north-south (Korea to Indonesia) and east-west (Japan to India) pattern of genetic differentiation [61]; our results partially support the pattern of north-south as in Battaglia et al. [61].

Overall, we observed significant population structure in *Ae. albopictus* (*F*_*ST*_ = 0.43, *P* ≤ 0.001). Similar results were observed in Zhong et al. [31] and Maynard et al. [75]. As mentioned, Zhong et al. [31] analyzed *cox*1 of *Ae. albopictus* from China, Taiwan, Japan, Singapore, Italy and the USA. Maynard et al. [75] using both microsatellite and mitochondrial markers observed significant relationship between genetic variability and geographical distance, but weak correlation in *Ae. albopictus* of Indo-Pacific regions.

Laotian *Ae. albopictus* populations were found to be very genetically related to the tropical Thailand strain. An allozyme study suggested that populations of *Ae. albopictus* in the eastern USA possibly originated from temperate Asian regions [67], while mtDNA variations revealed that populations in Represa do Congo and Sao Luis in Brazil formed a lineage paraphyletic to tropical Southeast Asian lineages, such as Cambodia, Vietnam, Thailand [52, 76] and likely Lao PDR.

## Conclusions

To our knowledge, this study represents the first genetic analysis of *Ae. albopictus* in Lao PDR. Laotian *Ae. albopictus* are genetically related to populations from tropical/subtropical regions. The high polymorphism but shallow population structure across Lao PDR and signs of a recent population expansion in *Ae. albopictus* may be the result of recent economic development that facilitates human-mediated movement of *Ae. albopictus*. We suggest that extensive movement and likely common reintroductions of *Ae. albopictus* to treated sites represent a major challenge to dengue control in Lao PDR.

## Supplementary information

**Additional file 1: Table S1.** Pairwise differentiation, *F*_*ST*_, among populations of *Ae. albopictus*. *Abbreviations*: China: GZ, Guangzhou; XM, Xiamen; JS, Jiangsu; Taiwan: TW, Xinzhu; Japan: JP, Nagazaki; Singapore: SG, Helios Block; Italy: IT, Trentino; USA: LA01, California; LA11, California; NJ, New Jersey; TX, Texas; HW, Hawaii; Lao PDR: BK, Borikhamxay; BO, Bokeo; CH, Champasak; KM, Khammuane; LN, Luangnamtha; LP, Luang Prabang; VC, Vientiane Capital; XB, Xayabouly; Thailand: TH, Thailand. ^**#**^Samples available in GenBank from Zhong et al. [31]. *Significant values after Bonferroni correction (*P* ˂ 0.05).

**Additional file 2: Figure S1.** Fixation indices obtained by SAMOVA for the best-clustering option at each pre-defined values of *K*. *Abbreviations*: *F*_*CT*_, variation among groups of populations; *F*_*SC*_, variation among populations within groups; *F*_*ST*_, variation among population among groups.

**Additional file 3: Table S2.** Geographical distances (in km) among *Ae. albopictus* from Lao PDR. *Abbreviations*: BK, Borikhamxay; BO, Bokeo; CH, Champasak; KM, Khammuane; LN, Luangnamtha; LP, Luang Prabang; VC, Vientiane Capital; XB, Xayabouly.

**Additional file 4: Figure S2.** Mismatch distributions showing the frequencies of pairwise differences of *Ae. albopictus* in Lao PDR.

**Additional file 5: Table S3.** Assignment of the Bayesian clustering analysis of *Ae*. *albopictus* populations. *Abbreviations*: China: GZ, Guangzhou; XM, Xiamen; JS, Jiangsu; Taiwan: TW, Xinzhu; Japan: JP, Nagazaki; Singapore: SG, Helios Block; Italy: IT, Trentino; USA: LA01, California; LA11, California; NJ, New Jersey; TX, Texas; HW, Hawaii—are samples from Zhong et al. [31]; Lao PDR: BK, Borikhamxay; BO, Bokeo; CH, Champasak; KM, Khammuane; LN, Luangnamtha; LP, Luang Prabang; VC, Vientiane Capital; XB, Xayabouly; Thailand: TH, Thailand. ^**#**^Samples available in Genbank from Zhong et al. [31]. The coefficient values above 0.25 are highlighted in bold.

## Data Availability

All data generated or analysed during the present study are included in this published article or available from the GenBank under the accession numbers MN080720-MN080765.
